# Laparoscopic cholecystectomy for acute cholecystitis: early or delayed? Evidence from a systematic review of discordant meta-analyses

**DOI:** 10.1097/MD.0000000000003835

**Published:** 2016-06-10

**Authors:** Guo-Min Song, Wei Bian, Xian-Tao Zeng, Jian-Guo Zhou, Yong-Qiang Luo, Xu Tian

**Affiliations:** a Department of Nursing, Tianjin Hospital, Tianjin; b Southwest Hospital/Southwest Eye Hospital, Southwest Hospital, Third Military Medical University, Chongqing; c Center for Evidence-Based and Translational Medicine, Zhongnan Hospital, Wuhan University, Wuhan; d Department of Oncology, Affiliated Hospital to Zunyi Medical University, Zunyi, Guizhou; e Emergency Department, Sichuan Anyue County People's Hospital, Anyue, Sichuan; f Department of Nursing, Chongqing Cancer Institute, Chongqing, China.

**Keywords:** acute cholecystitis, Jadad algorithm, laparoscopic cholecystectomy, meta-analysis

## Abstract

The laparoscopic cholecystectomy (LC) is an important approach of treating acute cholecystitis and the timing of performing this given treatment is associated with clinical outcomes. Although several meta-analyses have been done to investigate the optimal timing of implementing this treatment, the conflicting findings from these meta-analyses still confuse decision-making. And thus, we performed this systematic review to assess discordant meta-analyses and generate conclusive findings to facilitate informed decision-making in clinical context eventually. We electronically searched the PubMed, Cochrane Library, and EMBASE to include meta-analysis comparing early (within 7 days of the onset of symptoms) with delayed LC (at least 1 week after initial conservative treatment) for acute cholecystitis through August 2015. Two independent investigators completed all tasks including scanning and appraising eligibility, abstracting essential information using prespecified extraction form, assessing methodological quality using Oxford Levels of Evidence and Assessment of Multiple Systematic Reviews (AMSTAR) tool, and assessing the reporting quality using Preferred Reporting Items for Systematic Reviews and Meta-Analyses (PRISMA), as well as implementing Jadad algorithm in each step for the whole process. A heterogeneity degree of ≤50% is accepted. Seven eligible meta-analyses were included eventually. Only one was Level I of evidence and remaining studies were Level II of evidence. The AMSTAR scores varied from 8 to 11 with a median of 9. The PRISMA scores varied from 19 to 26. The most heterogeneity level fell into the desired criteria. After implementing Jadad algorithm, 2 meta-analyses with more eligible RCTs were selected based on search strategies and implication of selection. The best available evidence indicated a nonsignificant difference in mortality, bile duct injury, bile leakage, overall complications, and conversion to open surgery, but a significant reduction in wound infection, hospitalization, and operation duration and improvement of the quality of life when compared early LC with delayed LC. However, number of work days lost, hospital costs, and patient satisfaction are warranted to be assessed further. With the best available evidence, we recommend early LC to be as the standard treatment option in treating acute cholecystitis.

## Abstract 2

Supplemental Digital Content is available in the text

## 1 Introduction

Acute cholecystitis is a potentially life-threatening condition, which affects >20 million Americans yearly and causes high economic burden around the world.^[1]^ Gallstones is the major contributor to acute cholecystitis.^[1]^ Laparoscopic cholecystectomy (LC) is an important approach for treating acute cholecystitis nowadays.^[2]^ Issued data indicated that approximately 917,000 and >50,000 LCs were annually performed to treat acute cholecystitis in the United States and England, respectively.^[3–7]^ Although LCs have been extensively performed to manage acute cholecystitis, the optimal timing of LC for this given condition is inconclusive.

Traditionally, given the higher rate of morbidity such as bile duct injury, leakage, and conversion to open surgery, the delayed LC (DLC), which is defined as at least 1 week after initial conservative treatment, is commonly adopted in treating acute cholecystitis.^[8]^ However, several clinical studies supported early LC (ELC) (within 7 days of the onset of symptoms) to treat acute cholecystitis.^[9–11]^ Although some systematic reviews with meta-analysis investigated the optimal timing of LC for patients with acute cholecystitis previously, a consistent and conclusive conclusion has not yet been obtained from these systematic reviews. For instance, 2 meta-analyses^[3,12]^ qualitatively supported no difference between ELC and DLC in terms of mortality, 1^[13]^ indicated no difference in both approaches for this outcome, and 2^[14,15]^ considered this given outcome, but the effects of both approaches in causing mortality were identified. Moreover, 4 meta-analyses^[3,12,14,16]^ indicated that DLC shortens the duration of operation; however, 2^[2,13]^ identified no difference between DLC and ELC in terms of this given outcome. Most importantly, these conflicting findings will confuse the informed decision-making. And thus, we performed this systematic review of discordant meta-analyses to further assess the effects of ELC for acute cholecystitis compared with DLC.

Two purposes of this systematic review of discordant meta-analyses are as follows: helping decision-maker to assess and interpret these discordant meta-analyses findings, and concluding the treatment recommendations based on the best available evidence for clinical practice.

### 1.1 Materials and methods

The Preferred Reporting Items for Systematic Reviews and Meta-analysis (PRISMA)^[17]^ and Cochrane Handbook for Systematic Review of Interventions^[18]^ were compiled to generate reliable and valid systematic review and meta-analysis results with high quality. No ethical approval and patient written informed consent are needed because all processes of the whole study were performed on the basis of previous information. All processes were conducted using Microsoft Word (Microsoft Corporation, Redmond, WA).

### 1.2 Study retrieval

Systematic reviews or meta-analyses comparing ELC versus DLC for patients with acute cholecystitis were electronically searched independently by 2 investigators in PubMed, Cochrane Library, and EMBASE databases up to August 2015. The terms “acute cholecystitis,” “laparoscopic cholecystectomy,” “LC,” “celioscopic cholecystectomy,” “systematic review,” and “meta-analysis” were applied to perform search process. We also manually checked the bibliographies of all eligible studies to include any potential study. No other restrictions were imposed in our study. All search information was summarized in electronic supplementary material (ESM)-search strategy file.

### 1.3 Selection criteria

Implementing this systematic review of overlapping meta-analyses, we aimed to solve the discordance among findings from meta-analyses comparing the ELC to DLC in acute cholecystitis. Consequently, meetings abstract, letter to the editor, correspondence, systematic reviews without meta-analysis, and meta-analysis including non-RCTs are excluded from our study.

### 1.4 Selecting meta-analyses

Two investigators were assigned to independently select studies from all potential citations based on checking title and abstract of all citations captured initially and reviewing full version obtained from initial check stage in accordance with selection criteria for our study. Any discrepancies between these 2 investigators were resolved based on discussion or consulting a third investigator until a consensus was reached.

### 1.5 Information abstraction

The essential information for this study were abstracted independently by 2 investigators from each eligible meta-analysis using prespecified standard information extraction forms (ESM-Table 1) based on Microsoft Excel, including first author name, publication year, retrieval database, study design of the original trial, number of included RCTs, level of evidence, additional statistical analysis method, heterogeneity level, and all outcomes of interest. Any disagreements between these 2 investigators were eliminated by inviting a third investigator to arbitrate.

### 1.6 Quality assessment

We assigned 2 independent investigators to assess the search methodology of all eligible systematic reviews with meta-analyses based on retrieval sources and any restrictions including publication status and language of the original trial. Moreover, we also assigned 2 investigators to evaluate independently the methodological quality of each meta-analysis included using the Oxford Levels of Evidence^[19]^ and the Assessment of Multiple Systematic Reviews (AMSTAR) Instrument.^[20]^ AMSTAR, consists of 11 items, is currently reported as a measurement tool with reliability, validity, and responsibility to assess the methodological quality of systematic review and (or) meta-analysis extensively.^[21,22]^ We also adopted the preferred reporting items for systematic review and meta-analyses (PRISMA) to assess the reporting quality of each eligible meta-analysis.^[17]^ Score of 1 will be recorded if corresponding information for an item was reported; in contrast, score of zero will be entered. Discussion is the method to resolve any discordance between 2 investigators at this stage.

### 1.7 Assessing the heterogeneity

We assessed the heterogeneity level for each outcome of interest in all eligible meta-analyses based on information abstracted. The purpose of assessing the heterogeneity is to explore whether the systematic reviewer evaluated and dealt appropriately the heterogeneity and whether associated subgroup analysis and (or) sensitivity analysis were formally performed. In accordance with the judgment criteria documented in Cochrane Handbook for Systematic Review of Interventions,^[18]^ an *I*^*2*^ of ≤50% is eligible for the desired limit in our study.

### 1.8 Implementing the Jadad decision algorithm

Jadad decision algorithm is the guide to interpreting discordant systematic reviews with meta-analysis.^[23]^ To help decision-makers (including clinicians, policy-makers, researchers, and patients, depending on the context) make choices among alternative health care interventions when experts and the results of trials disagree, Jadad et al^[23]^ summarized the disagreement among systematic reviews with meta-analysis and 6 reasons including defining clinical question, specifying study inclusion and exclusion criteria, abstracting data, assessing the methodological quality, assessing the ability to pool studies, and summarizing the information were determined. Three investigators were assigned to implement the Jadad decision algorithm to identify which meta-analyses generated available best evidence on this topic until a consensus was reached.

## 2 Results

### 2.1 Search results and basic information of eligible meta-analyses

**Figure 1:**
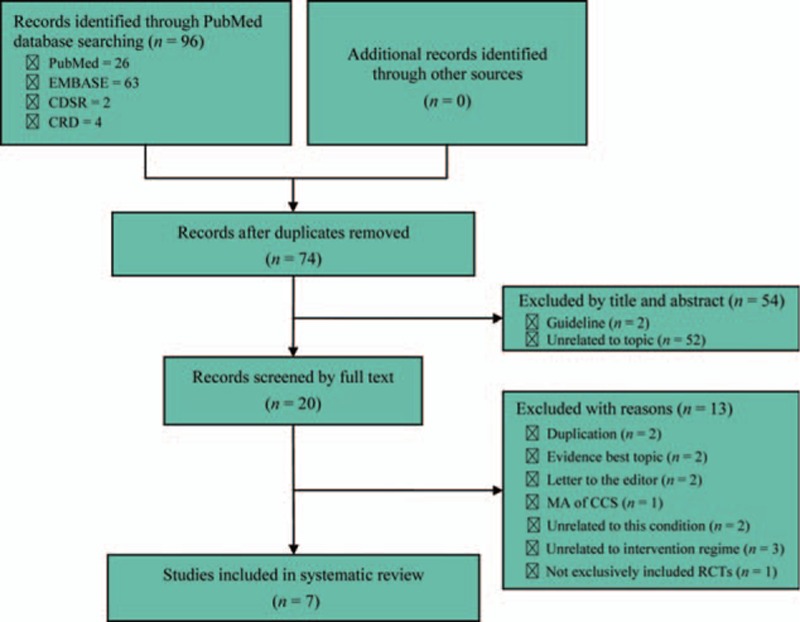
Flow diagram of identification and selection of meta-analyses: 95 potential records were initially captured using specified search terms and 7 were finally incorporated into this study based on comprehensive screened. CCS, case-control study; CDSR, Cochrane Database of Systematic Review; CRD, Center for Review and Dissemination; MA, meta-analysis; RCTs, randomized controlled trials.

We captured 96 records using specified search terms at the initial search stage and no citation was identified through other sources. The EndNote (version X7.1) literature management software was used to manage and scan the potential citations. After comprehensively reviewing all potential citations based on title, abstract, and full-version, 7 systematic reviews with meta-analyses^[2,3,12–16]^ were incorporated into our study to generate treatment recommendations. The flow diagram of identification and selection of meta-analysis and references bibliography were recorded in Fig. 1. The publication year of all eligible meta-analyses ranged from 2004 to 2015. The number of primary studies incorporated into each eligible meta-analysis varied from 3 to 16. The basic information of all meta-analyses included and the number of original studies incorporated into them were sorted in Tables 1 and 2, respectively.^[2,3,12–16]^

**Table 1:**
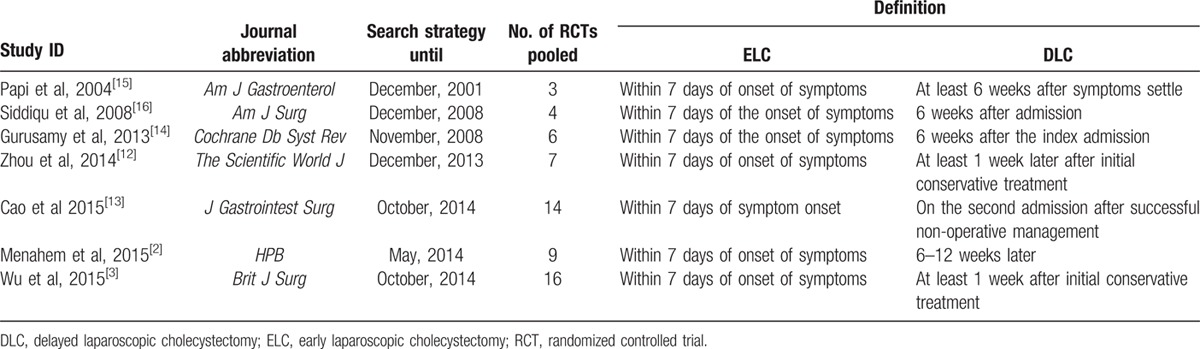
Basic information of each eligible meta-analysis.

**Table 2:**
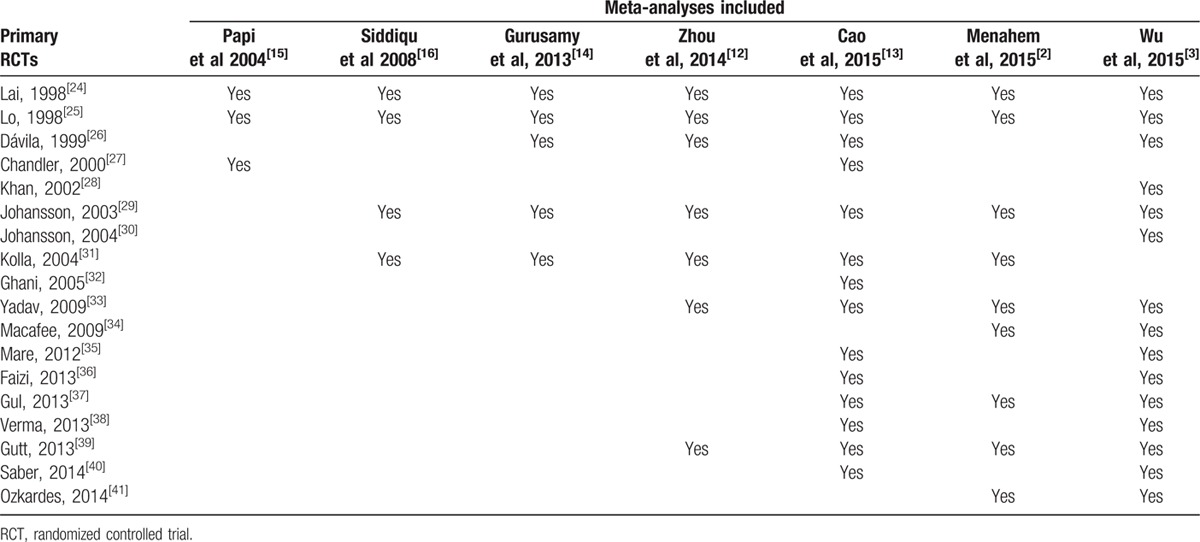
Primary RCTs incorporated into each eligible meta-analysis.

### 2.2 Search methodology

Comprehensive literature search is the fundamental guarantee to decrease the publication and time-lag bias. We assessed the search methodology quality of all eligible meta-analyses. The meta-analysis conducted by Menahem et al^[2]^ did not search the unpublished literature and restricted the publication language to English. Four meta-analyses^[3,13,14,16]^ did not impose the restriction of publication language. Remaining 2 meta-analyses^[12,15]^ did not provide the information of publication language. Two meta-analyses^[14,16]^ did not restrict the publication status of the original study and 2^[2,13]^ restricted the eligible study to trials. All meta-analyses searched the PubMed/Medline, EMBASE, and the Cochrane Library. The detail of search was described in Table 3.^[2,3,12–16]^

**Table 3:**
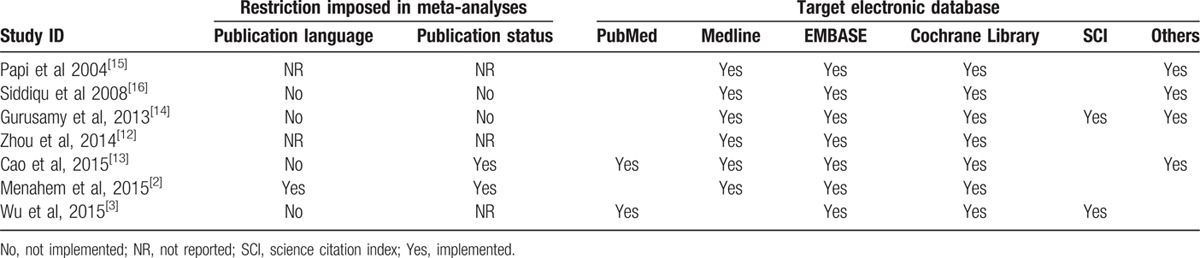
Search Methodology used by each meta-analysis.

### 2.3 Methodological quality

The reliability and stability of meta-analysis findings were generated from strict design and standard reporting. Level of evidence and methodology quality of all eligible meta-analyses were rated and assessed using Oxford Levels of Evidence^[19]^ and AMSTAR^[20]^ tool, respectively. The reporting quality was analyzed using the PRISMA.^[17]^

Based on Oxford Levels of Evidence, only 1 meta-analysis was regarded to include the Level I evidence and remaining meta-analyses included level of evidences I to II. Most of the meta-analyses^[2,3,12,14]^ used to Review Manager software to pool data. Only 1 meta-analysis performed Grading for Recommendation, Assessment, Development, and Evaluation (GRADE) to rate the level of evidence.^[14]^ Two meta-analyses used the trial sequential analysis (TSA) to calculate the required information size for each outcome of interest.^[3,14]^ Subgroup was used in 3 meta-analyses,^[13,14,16]^ sensitivity analysis was used in 4,^[2,12–14]^ and 2 meta-analyses reported information of register.^[21,23]^ You can find the detail on methodology quality in Table 4.^[2,3,12–16]^ The AMSTAR scores varied from 8 to 11 with a median of 9. Table 5 recorded the data of AMSTAR of each meta-analysis included and Table 6 presented the information of PRISMA.^[2,3,12–16]^

**Table 4:**
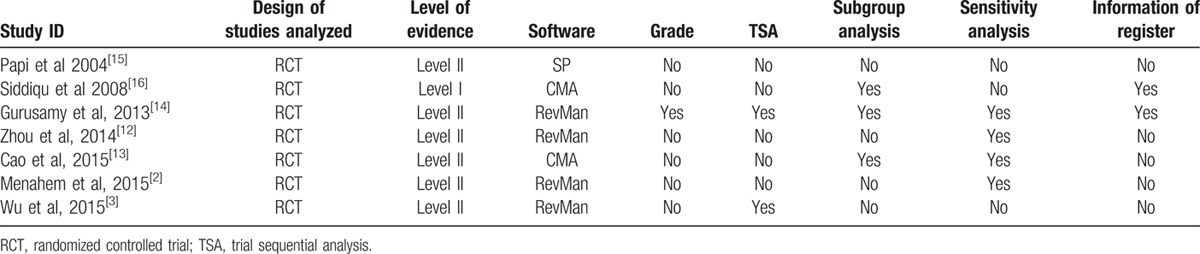
Methodological information for each included study.

**Table 5:**
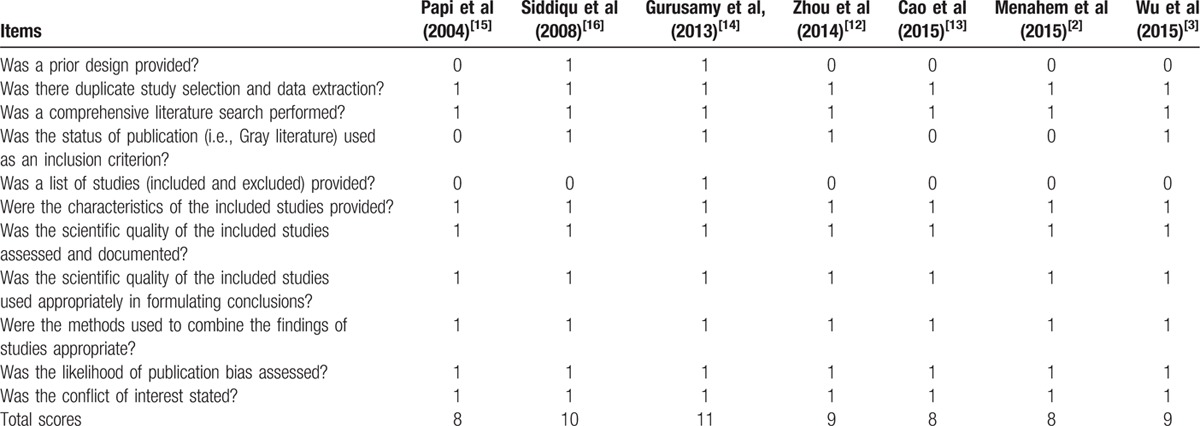
AMSTAR criteria for each included study.

**Table 6:**
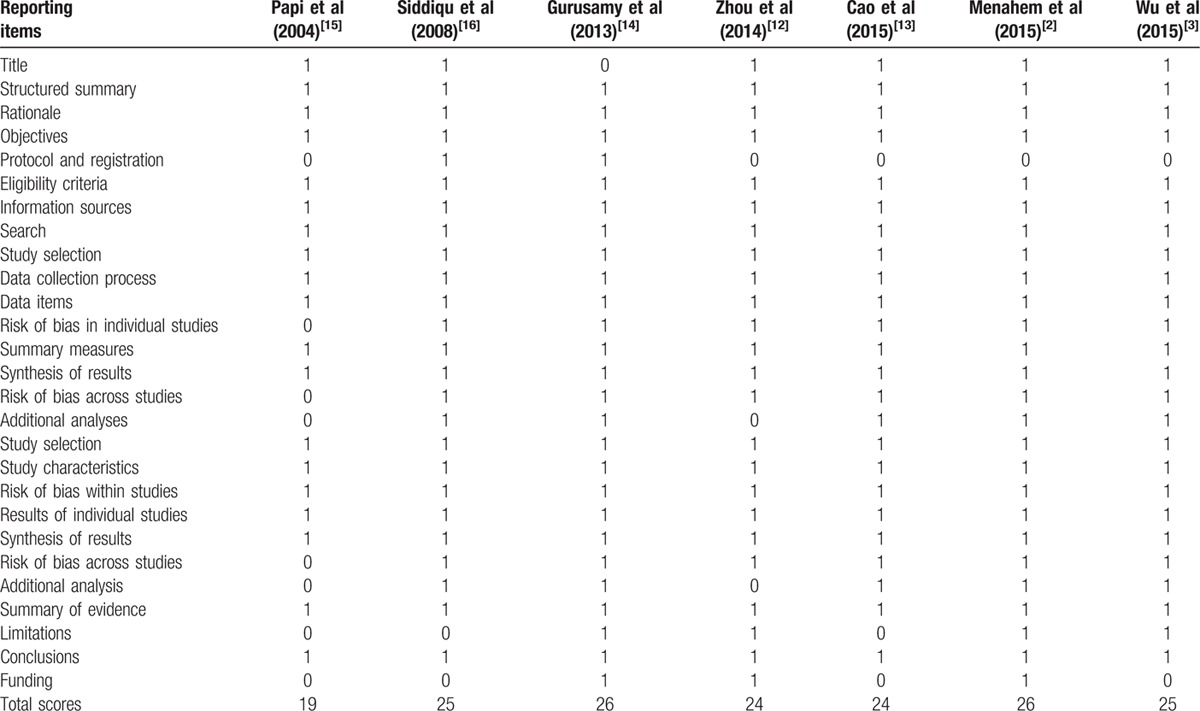
PRISMA criteria for each included study.

### 2.4 Assessing the heterogeneity

All eligible meta-analyses tested the heterogeneity level using *χ*^2^ test, *Q* test, or *I*^*2*^ statistic. We also summarized the heterogeneity level, subgroup analysis, and sensitive analysis of each meta-analysis in ESM-Table 2. Heterogeneity levels of most outcomes fulfilled the acceptable criteria (≤50%) released by the Cochrane Collaboration.^[18]^

### 2.5 Results of Jadad decision algorithm

**Figure 2:**
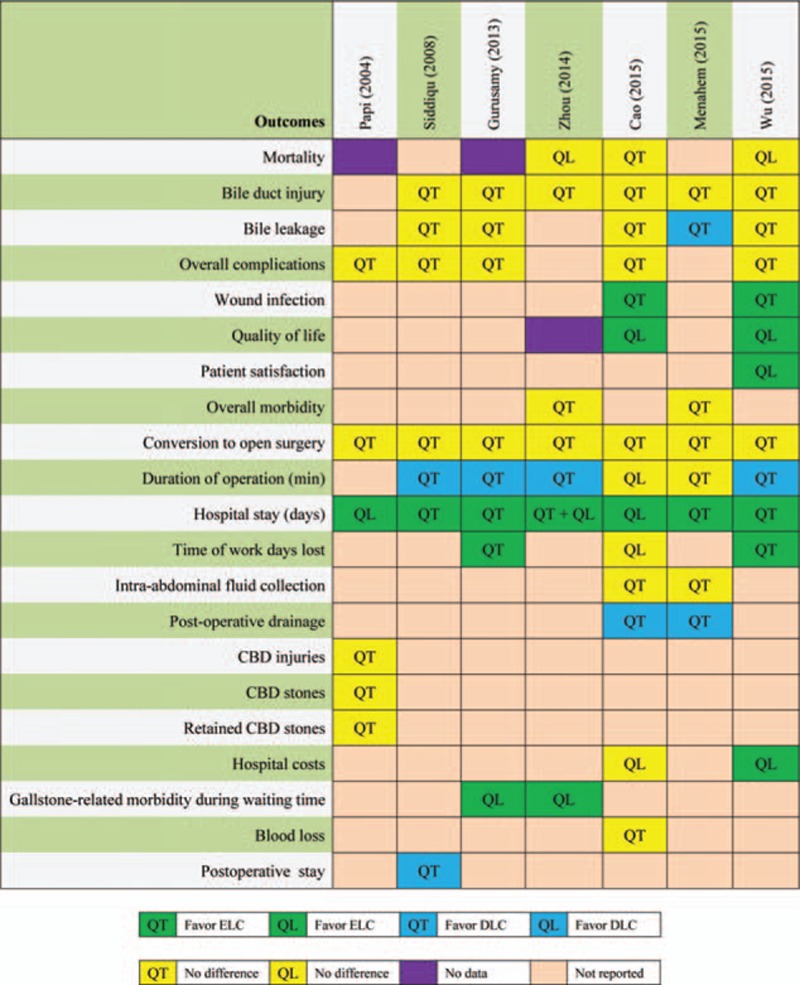
Results of all outcome measures reported in eligible meta-analyses. CBD, common bile duct; DLC, delayed laparoscopic cholecystectomy (at least 1 week after initial conservative treatment); ELC, early laparoscopic cholecystectomy (within 7 days of onset of symptoms); QL, qualitative analysis; QT, quantitative analysis.

**Figure 3:**
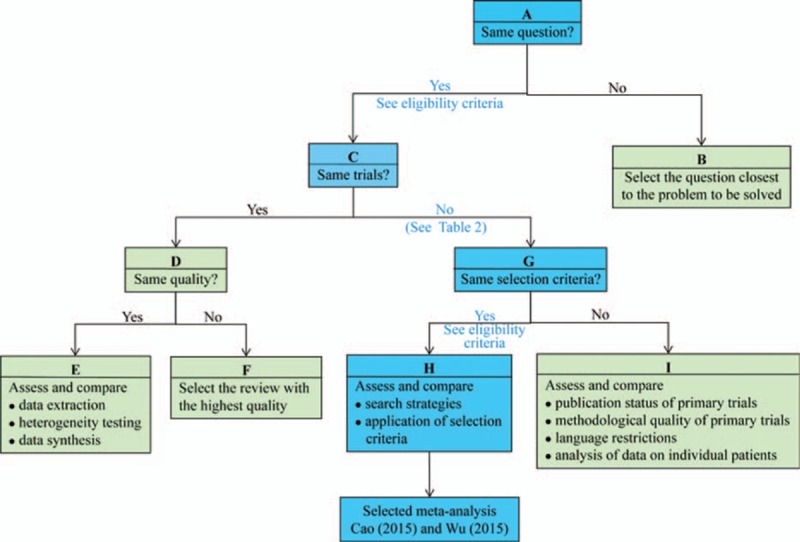
Flow diagram of Jadad decision algorithm. The square frame filled with light blue represented the decision process of the present study.

To visually assess all pooled results, we developed Fig. 2 to present all results of outcomes reported in all eligible meta-analyses. Given these criteria written in Jadad decision algorithm, we consequently selected 2 meta-analyses with more RCTs completed by Cao et al^[13]^ and Wu et al,^[3]^ respectively, based on 2 judgment factors including search strategy and application of selection criteria owing to all of meta-analyses addressed same clinical question, did not included the same primary trials, and employed similar selection criteria (Fig. 3). Two meta-analyses consistently indicated not significantly different in mortality, bile duct injury, bile leakage, overall complications and conversion to open surgery when ELC versus DLC for patients with acute cholecystitis. Both of them suggested that ELC effectively reduced wound infection, increased the quality of life, and reduced the length of hospitalization and the conclusion on hospital stay was confirmed by TSA in Wu et al's study. However, these 2 meta-analyses generated discordant conclusion on duration of operation, time of work days lost, and hospital costs. A point must be noted is that Wu et al performed a TSA to confirm the conclusion of which DLC spent less time to complete cholecystectomy than ELC. Moreover, this meta-analysis conducted by Wu et al indicated high patient satisfaction occurred in ELC group (Fig. 2).

## 3 Discussion

Systematic review or meta-analysis including all available original trials was listed to be the best available evidence source.^[42]^ Decision-makers including researchers, policy makers, and practitioners usually adopt a systematic review with meta-analysis to resolve those problems which cannot be solved in the original study and provide recommendations for informed decision-making eventually.^[43]^ However, discordant systematic reviews with meta-analysis on the same topic make the decision tend to be more complicated rather than simpler.^[23]^

For the purpose of facilitating informed decision-making, Jadad et al determined all potential sources which caused the discordance among meta-analyses in 1997. They summarized these sources as follows: clinical question which refers to populations of patients, interventions, outcome measures and settings, study selection and inclusion which consists of selection criteria, application of the selection criteria and strategies to search the literature, data extraction which can be divided into methods to measures outcomes, end points, and human error (that is random or systematic), assessment of study quality which can be performed based on methods to assess quality, interpretations of quality assessments and methods to incorporate quality assessments in review, assessment of the ability to combine studies which can be completed through statistical methods and clinical criteria to judge the ability to combine studies, and statistical methods for data synthesis.^[23]^

After implementing the Jadad decision algorithm, 2 meta-analyses performed by Cao et al^[13]^ and Wu et al^[3]^ were selected to generate treatment recommendations on timing of LC in treating acute cholecystitis based on the best available evidence and facilitate informed decision-making in clinical context eventually. The meta-analysis conducted by Cao et al^[13]^ suggested that incidence of wound infections was decreased, total length of hospitalization was shortened, and cost was decreased after ELC was implemented in acute cholecystitis; however, no differences in morbidity, bile duct injury, bile leakage, and conversion to open surgery were detected. As a result, these authors concluded that ELC should be recommended to be the standard treatment option for acute cholecystitis. Wu et al^[3]^ demonstrated that ELC lowers the risk of wound infection, shortens the hospital stay, increases the cost-effectiveness, duration of operation, patients satisfaction, and quality of life (QoL). Moreover, these authors obtained a statistical finding which ELC decreased the work days lost of patients with acute cholecystitis. However, authors acknowledged these findings from their study should be cautiously interpreted because of several limitations existed. For example, the conclusion of hospital costs generated from limited data and several eligible studies were rated to be at high risk of bias. Most importantly, Wu et al^[3]^ applied the TSA method to confirm the duration of operation and hospital stay. On the basis of findings from meta-analyses selected, we concluded that ELC can be implemented to decrease the incidence of wound infection and hospital stays, and to increase the QoL and patient satisfactions. It is important to note that ELC increased the duration of operation.

We strictly performed this systematic review of overlapping meta-analyses, but some limitations may impair the power of our findings. First, although a comprehensive literature search was conducted to include eligible as more adequate as possible, additional potential meta-analyses on this topic may be identified if the target databases were extended. Second, only meta-analyses including RCTs comparing the ELC with DLC in acute cholecystitis were eligible for these inclusion criteria designed in our systematic review, but several original RCTs incorporated into these 2 eligible meta-analyses have high risk of bias, which generate overestimated benefit and harm of ELC. Third, outcomes reported in these meta-analyses included are the subset of the universal set of outcomes of interest and thus the comprehensiveness of treatment recommendations from our systematic review will be limited.

## 4 Conclusion

With the best available evidence, we recommend ECL to be the standard treatment option in treating acute cholecystitis. However, the potential of ELC for working days lost and hospital costs are needed to be investigated further because of controversial conclusions existed in these 2 meta-analyses. Further, future studies should clearly define the definition of ELC and elective LC to provide more feasible and accurate information for clinical practice.

## Supplementary Material

Supplemental Digital Content

## Supplementary Material

Supplemental Digital Content

## Supplementary Material

Supplemental Digital Content
