# Supplementary material for: Laparoscopic cholecystectomy for acute cholecystitis: early or delayed? Evidence from a systematic review of discordant meta-analyses
Source: Medicine (Baltimore). 2016 Jun 10;95(23):e3835. doi: 10.1097/MD.0000000000003835 (PMC4907666; doi:10.1097/MD.0000000000003835)
Supplement: Supplemental Digital Content [file medi-95-e3835-s001.pdf]

Appendix.1 Search strategy of PubMed

Search	Query	Items found	Time
#8	Search ((((("Cholecystitis, Acute"[Mesh]) OR acute cholecystitis[Title/Abstract])) AND (("Cholecystectomy, Laparoscopic"[Mesh]) OR ((Celioscopic Cholecystectom*[Title/Abstract]) OR Laparoscopic cholecystectom*[Title/Abstract]))) AND ((((((systematic review[Title/Abstract]) OR meta-analys*[Title/Abstract]) OR meta analys*[Title/Abstract]) OR pooled analys*[Title/Abstract]) OR synthesis analys*[Title/Abstract]) OR synthesized analys*[Title/Abstract])	26	22:24:28
#7	Search (((((systematic review[Title/Abstract]) OR meta-analys*[Title/Abstract]) OR meta analys*[Title/Abstract]) OR pooled analys*[Title/Abstract]) OR synthesis analys*[Title/Abstract]) OR synthesized analys*[Title/Abstract]	167022	22:21:55
#6	Search ("Cholecystectomy, Laparoscopic"[Mesh]) OR ((Celioscopic Cholecystectom*[Title/Abstract]) OR Laparoscopic cholecystectom*[Title/Abstract])	13186	22:19:56
#5	Search (Celioscopic Cholecystectom*[Title/Abstract]) OR Laparoscopic cholecystectom*[Title/Abstract]	10679	22:19:27
#4	Search "Cholecystectomy, Laparoscopic"[Mesh]	10116	22:17:45
#3	Search ("Cholecystitis, Acute"[Mesh]) OR acute cholecystitis[Title/Abstract]	5523	22:16:04
#2	Search acute cholecystitis[Title/Abstract]	5233	22:13:33
#1	Search "Cholecystitis, Acute"[Mesh]	934	22:12:05

Appendix.2 Search strategy of Cochrane Library

Search Name: Laproscopic Cholecystectomy for Acute Cholecystitis

ID Search

#1 "acute cholecystitis":ti,ab,kw (Word variations have been searched)

#2 MeSH descriptor: [Cholecystitis, Acute] explode all trees

#3 #1 or #2

#4 Celioscopic Cholecystectom*:ti,ab,kw or Laparoscopic cholecystectom*:ti,ab,kw (Word variations have been searched)

#5 MeSH descriptor: [Cholecystectomy, Laparoscopic] explode all trees

#6 #4 or #5

#7 "systematic review":ti,ab,kw or "meta-analysis":ti,ab,kw or "meta-analyses":ti,ab,kw or pooled analys*:ti,ab,kw or synthesis analys*:ti,ab,kw (Word variations have been searched)

#8 MeSH descriptor: [Meta-Analysis] explode all trees

#9 #7 or #8

#10 #3 and #6 and #9

Appendix.3 Search strategy of EMBASE

No.	Query	Results
#15	**#3** AND **#7** AND **#14**	**63**
#14	**#8** OR **#9** OR **#10** OR **#11** OR **#12** OR **#13**	**217044**
#13	**'synthesis analysis'**	**325**
#12	**'pooled analysis'**	**7062**
#11	**'meta analysis (topic)'**/exp OR **'meta analysis (topic)'**	**21166**

#10	**'meta analysis'**/exp OR **'meta analysis'**	**141565**
#9	**'systematic review (topic)'**/exp OR **'systematic review (topic)'**	**12353**
#8	**'systematic review'**/exp OR **'systematic review'**	**126454**
#7	**#4** OR **#5** OR **#6**	**39493**
#6	**'cholecystectomy'**/exp	**39492**
#5	**'laparoscopic cholecystectomy'**	**13645**
#4	**'celioscopic cholecystectomy'**	**21**
#3	**#1** OR **#2**	**8151**
#2	**'acute cholecystitis'**/exp	**4898**
#1	**'acute cholecystitis'**/exp OR **'acute cholecystitis'**	**8151**
