# Supplementary material for: Laparoscopic cholecystectomy for acute cholecystitis: early or delayed? Evidence from a systematic review of discordant meta-analyses
Source: Medicine (Baltimore). 2016 Jun 10;95(23):e3835. doi: 10.1097/MD.0000000000003835 (PMC4907666; doi:10.1097/MD.0000000000003835)
Supplement: Supplemental Digital Content [file medi-95-e3835-s003.pdf]

ESM-TABLE 2. I^2 statistic or χ^2 value of each outcome, subgroup and sensitive analysis

Items	Papi (2004)	Siddiqu (2008)	Gurusamy (2013)	Zhou (2014)	Cao (2015)	Menahem (2015)	Wu (2015)
Mortality			NP	NP	0.0%		
Bile duct injury		NR	28.0%	28.0%	NR	NR	0.0%
Low risk of bias			51.0%				
High risk of bias			28.0%				
Less than 4 days			0.0%				
Less than 7 days			0.0%				
At least 50 LC or consultant			52.0%				
Trainees without 50 LC			28.0%				
Best-best scenario			25.0%				
Best-worst scenario			49.0%				
Worst-best scenario			24.0%				
Worst-worst scenario			18.0%				
Bile leakage		NR			0.0%	0.0%	0.0%
Overall complications	2.42	4.46	0.0%		50.8%		67.0%
Low risk of bias			0.0%				
High risk of bias			0.0%				
Less than 4 days			0.0%				
Less than 7 days			22.0%				
At least 50 LC or consultant			0.0%				
Trainees without 50 LC			0.0%				
Best-best scenario			0.0%				
Best-worst scenario			55.0%				
Worst-best scenario			0.0%				
Worst-worst scenario			35.0%				
Wound infection					0.0%		0.0%
Quality of life					NP		NP
Patient satisfaction							NP
Overall morbidity				56.0%		NR	
Conversion to open surgery	0.68	1.84	0.0%	0.0%	NR	NR	
Low risk of bias			0.0%				
High risk of bias			13.0%				
Less than 4 days			10.0%				
Less than 7 days			0.0%				

Continued ESM-TABLE 2

ESM-TABLE 2. I^2 statistic or χ^2 value of each outcome, subgroup and sensitive analysis

Items	Papi (2004)	Siddiqu (2008)	Gurusamy (2013)	Zhou (2014)	Cao (2015)	Menahem (2015)	Wu (2015)
At least 50 LC or consultant			0.0%				
Trainees without 50 LC			0.0%				
Best-best scenario			0.0%				
Best-worst scenario			45.0%				
Worst-best scenario			0.0%				
Worst-worst scenario			7.0%				
Duration of operation (min)		0.44	76.0%	76.0%	NP	NR	30.0%
Low risk of bias			72.0%				
High risk of bias			0.0%				
Less than 4 days			0.0%				
Less than 7 days			84.0%				
At least 50 LC or consultant			0.0%				
Trainees without 50 LC			77.0%				
Hospital stay (days)	NP	2.05	0.0%	0.0%	NP	92.0%	76.0%
Low risk of bias			0.0%				
High risk of bias			0.0%				
Less than 4 days			0.0%				
Less than 7 days			0.0%				
At least 50 LC or consultant			0.0%				
Trainees without 50 LC			0.0%				
Time of work days lost			0.0%		NP		52.0%
Intra-abdominal fluid collection					NR	NR	
Post-operative drainage						NR	
CBD injuries	0.00						
CBD stones	1.00						
Retained CBD stones	0.00						
Hospital costs					NP		NP
Gallstone-related morbidity during waiting time				NP			
Blood loss					NR		
Use of sub-hepatic drain					NR		

CBD, common bile duct; NP, not pooled; NR, not reported.
